# Editorial: Neural mechanisms of sensory-motor integration in manual tasks

**DOI:** 10.3389/fpsyg.2026.1982150

**Published:** 2026-09-22

**Authors:** Mattia Marangon, Piotr Styrkowiec, Ivilin Peev Stoianov

**Affiliations:** 1 Department of Neuroscience, Biomedicine and Movement, University of Verona, Verona, Italy; 2 Institute of Psychology, University of Wrocław, Wrocław, Poland; 3 Institute of Cognitive Sciences and Technologies (ISTC), National Research Council, Padua, Italy

**Keywords:** manual actions, motor control, neural plasticity, predictive processing, sensorimotor learning, sensory-motor integration

For much of the history of psychology and neuroscience, the brain was commonly conceived as an input-output system that receives information from the environment, constructs internal representations of the world, and generates appropriate motor commands. This classical picture has progressively evolved, echoing ideas first anticipated by von Helmholtz (1924) more than a century ago. Contemporary cognitive neuroscience increasingly views the brain as a homeostatic organ, whose primary function is not to faithfully interpret the external world, but to maintain the organism's integrity through adaptive interactions with its environment (Friston, 2010). Within this framework, neural processing is fundamentally predictive: the brain relies on internal generative models to infer the hidden causes of sensory signals, predict the sensory consequences of action, anticipate future states of the body and the environment, and continuously update these models by minimizing prediction errors (Rao and Ballard, 1999).

The predictive perspective has also reshaped theories of motor control (Franklin and Wolpert, 2011; Floegel et al., 2023; Priorelli et al., 2024). Rather than issuing motor commands reactively in response to sensory input, the brain is thought to prepare action plans in advance through distributed fronto-parietal networks and to continuously refine them as movements unfold and environmental conditions change (Cisek and Kalaska, 2010; Michalski et al., 2020; Li et al., 2022). These ideas have inspired a new generation of neurocomputational models, including control-as-inference frameworks (Priorelli et al., 2023) and end-to-end embodied hybrid architectures that integrate high-level decision-making with low-level sensorimotor control (Priorelli and Stoianov, 2025; Priorelli et al., 2025). Such models combine top-down prior beliefs with state estimates inferred from multiple sensory modalities—including exteroceptive, proprioceptive, and interoceptive signals—to generate smooth, efficient, and adaptive behavior in both open and closed-loop conditions (Adams et al., 2012). However, these theoretical developments require increasingly rich empirical evidence spanning behavior, neurophysiology, and computational modeling.

A central challenge is therefore to identify the neural and behavioral mechanisms through which these internal generative models are acquired, represented, updated, and ultimately translated into coordinated action. The studies collected in this Frontiers Research Topic, *Neural Mechanisms of Sensory-Motor Integration in Manual Tasks*, contribute to this effort by investigating the neural basis of sensory-motor integration in manual behavior from complementary experimental and theoretical perspectives. Although the contributions differ substantially in methodology and scope, many converge on a common principle: successful motor behavior depends not simply on constructing increasingly accurate representations of the external world, but on developing internal models that support efficient, adaptive, and goal-directed action. Using multidisciplinary approaches combining computational modeling, behavioral experimentation, neurophysiological recordings, and clinical investigations, these studies illustrate how the nervous system selectively exploits information that is most relevant for achieving functional behavioral outcomes.

One illustration comes from perceptual expertise. Expertise is often associated with increasingly accurate perception, yet the work of Hao and Chen suggests a more nuanced picture. Expert calligraphers exhibited a systematic dissociation between the force they objectively produced and the effort they subjectively experienced, indicating that long-term practice reshapes internal sensorimotor models according to task-specific functional and aesthetic demands. A related phenomenon emerged in children with cerebral palsy, where affective touch—but not generic tactile stimulation—improved movement control and produced measurable changes in EEG activity (Sabater-Gárriz et al.). In both studies, behavior appeared to benefit from selectively weighting information that was most relevant for successful action, rather than from veridical representations of sensory inputs.

Several contributions further demonstrate the flexibility of motor coordination. Tool-use behaviors reorganized according to task complexity, handedness, and processing demands (Przybylski and Kroliczak), while aging reduced the functional specialization required for asymmetric bimanual coordination (Kiani et al.). Nevertheless, substantial plasticity remained evident. Bilateral transcranial direct current stimulation over the motor cortex enhanced coordination performance (Lee et al.), whereas even a brief mindfulness training improved movement stability by strengthening inhibitory control (Devillers-réolon et al.). Together, these findings highlight the remarkable capacity of sensorimotor networks to reorganize and adapt across a wide range of behavioral contexts and interventions.

The efficient organization of sensory processing also emerged as an important theme. During three-dimensional navigation, participants performed more effectively when spatial information was distributed across complementary sensory modalities and referenced to stable spatial frames (Suzuki et al.). Rather than processing all available information uniformly, the nervous system appears to exploit specialized computational pathways that minimize processing demands while preserving behavioral performance.

The close coupling between cognitive and motor processes is equally evident in handwriting, where movement speed and legibility depend on the coordinated contribution of executive functions, memory, and motor control (Frikha et al.). Finally, the adaptability of sensorimotor systems was shown to extend to clinical populations. Individuals with Complex Regional Pain Syndrome retained the ability to adapt their movements despite persistent chronic pain (Dagenais et al.), suggesting that sensorimotor learning mechanisms continue to refine behavior even under severe physiological constraints.

Taken together, the contributions to this Research Topic reinforce the emerging view that sensorimotor behavior is guided by adaptive internal models that are continuously refined through interaction with the environment. From the perspective of predictive processing, expertise, learning, and rehabilitation may therefore be understood less as the progressive construction of increasingly detailed representations of the external world than as the continuous optimisation of internal models that support effective action under changing environment and physiological conditions throughout the lifespan.

Although many important questions remain open—including the precise neural mechanisms through which predictive internal models are formed, updated, and coordinated across multiple levels of the sensorimotor hierarchy—the studies collected here provide valuable experimental evidence and methodological advances that help bridge computational theories with empirical neuroscience. We hope that this Research Topic will stimulate further discussion, foster new interdisciplinary collaborations, and inspire future investigations into the neural principles that give rise to adaptive sensorimotor behavior.
